# Effect of Esketamine Added to Propofol Sedation on Desaturation and Hypotension in Bidirectional Endoscopy: A Randomized Clinical Trial

**DOI:** 10.1001/jamanetworkopen.2023.47886

**Published:** 2023-12-20

**Authors:** Nan Song, Yi Yang, Zhong Zheng, Wen-cheng Shi, Ai-ping Tan, Xi-sheng Shan, Hong Liu, Lingzhong Meng, Ke Peng, Fu-hai Ji

**Affiliations:** 1 Department of Anesthesiology, The First Affiliated Hospital of Soochow University, Suzhou, Jiangsu, China; 2 Institute of Anesthesiology, Soochow University, Suzhou, Jiangsu, China; 3 Department of Anesthesiology, The People’s Hospital of Suzhou New District, Suzhou, Jiangsu, China; 4 Department of Anesthesiology, Taicang First People’s Hospital, Taicang, Jiangsu, China; 5 Department of Anesthesiology and Pain Medicine, University of California Davis Health System, Sacramento; 6 Department of Anesthesia, Indiana University School of Medicine, Indianapolis

## Abstract

**Importance:**

Propofol sedation is widely used for endoscopic procedures, but it poses risks of hemodynamic and respiratory depression. The addition of esketamine as an adjuvant may reduce propofol requirements and associated adverse events.

**Objective:**

To evaluate the effects of low-dose esketamine added to propofol-based sedation on desaturation and hypotension during same-visit bidirectional endoscopy.

**Design, Setting, and Participants:**

This multicenter, double-blind, placebo-controlled randomized clinical trial assessed patients from 3 teaching hospitals in China who were scheduled for same-visit bidirectional endoscopy between February 8 and November 30, 2022, and randomly assigned to receive esketamine or normal saline (placebo).

**Interventions:**

After induction of sedation with 0.1 μg/kg of sufentanil and 0.5 mg/kg of propofol, patients in the esketamine group received 0.15 mg/kg of intravenous esketamine, whereas patients in the placebo group received an equivalent volume of saline. Sedation was achieved through propofol titration.

**Main Outcomes and Measures:**

The primary outcome was the composite of desaturation and hypotension during the procedures. Secondary outcomes included desaturation, hypotension, propofol requirements, postprocedure pain and fatigue, nausea or vomiting, dizziness or headache, hallucination or nightmare, endoscopist satisfaction, and patient satisfaction.

**Results:**

Among the 663 initially enrolled patients, 660 completed the study (median [IQR] age, 48 [36-57] years; 355 [53.8%] female), with 331 randomized to the esketamine group and 329 to the placebo group. The administration of esketamine compared with placebo significantly reduced the incidence of the composite outcome of desaturation and hypotension (8.2% vs 21.0%; difference, −12.8 percentage points; odds ratio [OR], 0.34; 95% CI, 0.21-0.54; *P* < .001). Additionally, esketamine led to significantly lower incidences of desaturation (OR, 0.36; 95% CI, 0.18-0.72; false discovery rate *q* = .01) and hypotension (OR, 0.33; 95% CI, 0.18-0.60; *q* < .001) and reduced propofol requirements (difference, −58.9 mg; 95% CI, −65.7 to −52.2 mg; *q* < .001), without significant effects on other secondary outcomes.

**Conclusions and Relevance:**

In this randomized clinical trial of patients undergoing same-visit bidirectional endoscopy, the administration of low-dose esketamine resulted in an approximately 61% reduction in the incidence of desaturation and hypotension, accompanied by decreased propofol requirements. These findings support the use of esketamine as an adjuvant to propofol-based sedation in endoscopic procedures.

**Trial Registration:**

Chinese Clinical Trial Registry Identifier: ChiCTR2200055938

## Key Points

**Question:**

Does low-dose esketamine added to propofol sedation reduce desaturation and hypotension during gastrointestinal endoscopic procedures?

**Findings:**

In this multicenter randomized clinical trial of 660 patients who underwent same-visit bidirectional endoscopy, the composite outcome of desaturation and hypotension occurred in 8.2% of patients who received low-dose esketamine, which was significantly lower than the 21.0% occurrence in patients who received normal saline.

**Meaning:**

The findings of this study support the incorporation of esketamine as an adjuvant to propofol sedation for patients undergoing same-visit bidirectional endoscopy.

## Teaser

This randomized clinical trial assesses whether esketamine as an adjuvant to propofol sedation leads to a reduced incidence of desaturation and hypotension during endoscopic procedures.

## Introduction

During the last decade, the volume of gastrointestinal endoscopic procedures has increased 10-fold.^1,2,3^ Many patients now undergo esophagogastroduodenoscopy and colonoscopy during the same hospital visit, commonly known as same-visit bidirectional endoscopy. To enhance patient comfort and facilitate these procedures, the use of sedation has become increasingly prevalent.^4,5^ Although propofol, either alone or in combination with analgesics, is the standard sedation choice for endoscopic procedures, it is not without its drawbacks, as adverse events, such as desaturation and hypotension, have been reported.^6,7,8^

One promising alternative is esketamine, an *N*-methyl-d-aspartate receptor antagonist and the dextrorotatory isomer of ketamine. Esketamine possesses twice the potency of ketamine in terms of hypnotic and analgesic effects, with fewer psychiatric adverse effects.^9,10,11^ Previous studies have also indicated that a subanesthetic dose of esketamine can maintain hemodynamic stability and reduce respiratory depression in surgical patients.^12,13,14^ This finding has prompted exploration of the use of low-dose esketamine as an adjuvant to propofol in providing sedation and analgesia to patients undergoing gastrointestinal endoscopic procedures. With this context in mind, we designed this multicenter randomized clinical trial to investigate the effects of adding low-dose esketamine to propofol-based sedation in patients undergoing same-visit bidirectional endoscopy. Our primary hypothesis was that esketamine as an adjuvant to propofol sedation leads to a reduced incidence of the composite outcome of desaturation and hypotension during these endoscopic procedures.

## Methods

### Study Design

This multicenter, double-blind, placebo-controlled randomized clinical trial was conducted at 3 medical centers in eastern China. Ethical approval was obtained from the ethics committees of all study centers. This study was registered on the Chinese Clinical Trial Registry before enrolling the first patient. All patients provided written informed consent. The study adhered to the principles outlined in the Declaration of Helsinki^15^ and followed the Consolidated Standards of Reporting Trials (CONSORT) reporting guideline. The rationale of this study was previously published.^16^ The study protocol and statistical plan are available in Supplement 1. There were no modifications to the protocol during the study implementation.

### Patients

Eligible patients were recruited from February 8 to November 30, 2022. Inclusion criteria were age of 18 to 70 years, American Society of Anesthesiologists (ASA) physical status I to II, body mass index (BMI) of 18 to 30 (calculated as weight in kilograms divided by height in meters squared), and same-visit bidirectional endoscopy scheduled. Exclusion criteria were severe cardiovascular or pulmonary diseases, kidney or liver dysfunction, neurocognitive or psychiatric disorders, seizures or epilepsy, alcoholism, preoperative use of sedatives or analgesics, or allergies to the medications under investigation.

### Randomization and Blinding

Research personnel who were not involved in patient recruitment, data collection, or outcome assessment performed the randomization (1:1, permuted blocks of 2 and 4, and stratified by trial sites) using an online tool.^17^ The randomization details were concealed within identical sealed opaque envelopes to maintain blinding. Shortly before patients were sedated, an independent nurse anesthetist who was unaware of the randomization opened the envelopes and assigned eligible patients to the esketamine group or the normal saline placebo group. The same nurse anesthetist prepared both esketamine and normal saline medications in indistinguishable syringes because both substances are clear and colorless solutions. For the entirety of the study, all participants, including patients, anesthesiologists, endoscopists, nurses, and outcome assessors, remained blinded to the randomization allocation.

### Periprocedural Care

Patients’ vital signs, including heart rate, oxygen saturation as measured by pulse oximetry (Spo_2_), and noninvasive blood pressure, were closely monitored using an anesthetic monitor (GE HealthCare). Before the commencement of the procedures, we measured patients’ blood pressure, Spo_2_, and heart rate and used these measurements as the baseline values. Spo_2_ was monitored with the probe attached to the left index finger, whereas blood pressure was measured at a 2-minute interval with the cuff placed around the right upper arm. To ensure adequate oxygenation, patients received nasal cannula oxygen supplementation at 3 L/min. The bidirectional endoscopic procedures were performed sequentially by experienced endoscopic teams, beginning with esophagogastroduodenoscopy and followed by colonoscopy. On completion of the procedures, patients were transferred to a designated recovery room for postprocedure care.

### Study Interventions

Both groups of patients were administered 0.1 μg/kg of intravenous sufentanil and 0.5 mg/kg of propofol to induce sedation. Subsequently, patients in the esketamine group were given 0.15 mg/kg of intravenous esketamine, whereas patients in the placebo group received an equivalent volume of saline. Throughout the procedures, sedation was adjusted by titrating propofol doses, typically 0.2 to 0.3 mg/kg, to achieve the predetermined sedation level. At the start of esophagogastroduodenoscopy, the targeted sedation level was set at a Modified Observer’s Assessment of Alertness/Sedation scale score of 1 (only responding to a trapezius squeeze stimulus).^18,19^ During the subsequent colonoscopy, the target sedation level was set at a score of 2 (only responding to prodding or shaking stimuli).

Desaturation was defined as Spo_2_ less than 90% for 10 seconds or longer. Interventions for desaturation included supplemental oxygen of 5 to 10 L/min and airway interventions (jaw extension, oral or nasal airway, or endotracheal intubation). Hypotension was defined as a systolic blood pressure less than 80 mm Hg or a decrease in systolic blood pressure greater than 30% of baseline. Interventions for hypotension included 5 mg of intravenous ephedrine or 50 μg of phenylephrine.

### Study Outcomes

The primary outcome measure was a composite of desaturation and hypotension (any event of desaturation, hypotension, or both) during sedation. Prespecified secondary outcomes included individual occurrences of desaturation and hypotension; total propofol dose; pain and fatigue levels at emergence from sedation and 15 minutes after the procedure; the occurrence of dizziness or headache, hallucination or nightmare episodes, and nausea or vomiting; and satisfaction of patients and endoscopists. Postprocedure pain or fatigue was evaluated using the numerical rating scale (range, 0-10, with 0 indicating no pain or fatigue and 10 indicating the most severe pain or fatigue experienced by the patient). Patient satisfaction and endoscopist satisfaction were measured using a 5-point Likert scale (range, 0-5, with a score of 1 indicating being very dissatisfied and a score of 5 indicating being highly satisfied).

### Data Collection

We collected patients’ demographic and baseline data. During sedation, patients’ vital signs were continuously captured by monitors, and the anesthesiologists documented anesthesia information on paper anesthesia records. Desaturation and hypotension events as well as interventions were also recorded. In the case of artifacts in the Spo_2_ measurement, a decision was made by the anesthesia team to ascertain whether it was a desaturation event or not. The raw data collected by the monitors and anesthesia records were reviewed by 2 independent researchers to confirm the occurrence of study outcomes. All data were entered in case report forms and registered in an electronic research database. Patient safety, trial implementation, and data management were monitored by an independent data and safety monitoring board. The database was locked following the completion of the last patient. The research data were sent to a statistician for analysis per the predefined protocol.

### Statistical Analysis

Previous studies have investigated desaturation and hypotension as separate outcomes during endoscopic procedures with propofol sedation combined with an opioid agent. Chiang et al^20^ reported an incidence of desaturation of 31% to 45%, and Yin et al^21^ reported a desaturation rate of 23% and hypotension of 17%. In a study by Eberl et al,^22^ 8 of 79 patients (10.0%) experienced desaturation and 17 (21.5%) developed hypotension. Nonetheless, none of those studies assessed the composite outcome of desaturation and hypotension. Hence, we performed a pilot study of 32 patients having the same procedures and sedation as the placebo group, showing that the composite outcome of desaturation and hypotension occurred in 10 patients (31.3%; 7 patients experienced desaturation, 5 developed hypotension, and 2 had both).^16^ We hypothesized that incorporating low-dose esketamine as an adjuvant to propofol could lower the incidence of the composite outcome from 30% to 20%. To achieve a statistical power of 80% with an α = .05, we calculated that each group would require 294 patients. Therefore, we recruited 660 patients (n = 330 in each group) to account for any potential dropouts.

For continuous variables, the Shapiro-Wilk test was used to assess the distribution. Normally distributed data were presented as means (SDs). Nonnormally distributed data were presented as medians (IQRs), and intergroup comparisons were performed using the Mann-Whitney *U* test. Categorical variables were expressed as numbers (percentages) and analyzed using the χ^2^ test or Fisher exact test, as appropriate. For the baseline data, standardized mean differences (SMDs) were provided as a measure of the imbalance between the groups. The treatment effects of esketamine vs placebo were evaluated using odds ratio (OR) or median difference (MD) with 95% CIs. We used multivariable logistic regression or linear regression to adjust the study outcomes for potential confounding factors, including age, BMI, smoking status, hypertension, and diabetes, as well as trial sites. Additionally, we performed regression analyses for the study outcomes, adjusting for covariates with an SMD greater than 0.10 (sex, hypertension, and procedure time) and trial sites, as well as for sex and trial sites. Prespecified subgroup analyses of the primary outcome were conducted based on trial sites, age, BMI, current smoking, history of hypertension, and history of diabetes.

Data were analyzed in the modified intention-to-treat population, encompassing all randomized patients who underwent endoscopic procedures with available primary outcome data. No interim analysis or imputation for missing data was performed. Statistical analyses were performed using R software, version 3.6.0 (R Foundation for Statistical Computing). For the primary outcome, the significance level was a 2-sided *P* < .05. For the secondary outcomes, multiple testing was adjusted using the Benjamini-Hochberg method, and the significance level of a false discovery rate *q* < .05 was applied.

## Results

**Figure 1.**
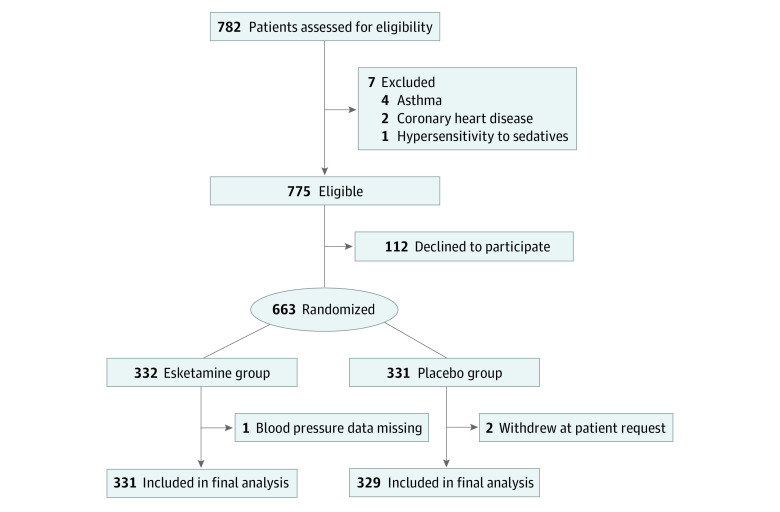
CONSORT Flow Diagram

Of 782 patients screened for eligibility, 663 (median [IQR] age, 48 [36-57] years; 355 [53.8%] female and 305 [46.2%] male; median [IQR] BMI, 23.2 [21.3-25.0]) were randomized. During the study, 1 patient in the esketamine group had missing data on blood pressure, and 2 patients in the placebo group withdrew their consent. Those 3 patients were the only ones with missing data on the primary outcome. Ultimately, data from 660 patients (331 in the esketamine group and 329 in the placebo group) were included and analyzed (Figure 1).

Demographic and baseline characteristics, as well as the procedure time, are presented in Table 1. There are imbalances between groups in sex, height and weight (related to sex), hypertension, and procedure time. There were 195 women (58.9%) in the esketamine group and 160 (48.6%) in the placebo group (SMD, 0.21). Forty-one patients (12.4%) in the esketamine group and 55 (16.7%) in the placebo group had hypertension (SMD, 0.12). The median (IQR) procedure time was 20 (16-25) minutes in the esketamine group and 18 (15-22) minutes in the placebo group (SMD, 0.15).

**Table 1.** Demographics, Baseline Data, and Procedure Time^a^

Characteristic	Esketamine (n = 331)	Placebo (n = 329)	SMD
Age, median (IQR), y	48 (36-57)	47 (37-57)	0.002
Sex			
Female	195 (58.9)	160 (48.6)	0.21
Male	136 (41.1)	169 (51.4)	
Height, median (IQR), m	1.65 (1.6-1.7)	1.67 (1.6-1.7)	0.21
Weight, median (IQR), kg	62 (55-70)	63 (57-72)	0.18
BMI, median (IQR)	22.9 (21.1-24.9)	23.2 (21.5-24.9)	0.08
ASA physical status			
I	257 (77.6)	242 (73.6)	0.10
II	74 (22.4)	87 (26.4)	
Current smoker	28 (8.5)	35 (10.6)	0.07
Hypertension	41 (12.4)	55 (16.7)	0.12
Diabetes	21 (6.3)	29 (8.8)	0.09
Baseline measurements			
SBP, median (IQR), mm Hg	129 (118-141)	128 (120-141)	0.005
DBP, mean (SD), mm Hg	75.4 (11.0)	75.7 (11.2)	0.03
MAP, mean (SD), mm Hg	93.7 (11.9)	93.9 (11.5)	0.02
HR, median (IQR), beats per min	76 (68-84)	75 (70-82)	0.03
Spo_2_, median (IQR), %	100 (99-100)	100 (99-100)	0.02
Trial sites			
Site 1	220 (66.5)	220 (66.9)	0.01
Site 2	56 (16.9)	54 (16.4)	
Site 3	55 (16.6)	55 (16.7)	
Procedure time, median (IQR), min	20 (16-25)	18 (15-22)	0.15

Abbreviations: ASA, American Society of Anesthesiologists; BMI, body mass index (calculated as weight in kilograms divided by height in meters squared); DBP, diastolic blood pressure; HR, heart rate; MAP, mean arterial pressure; SBP, systolic blood pressure; SMD, standardized mean difference; Spo_2_, oxygen saturation as measured by pulse oximetry.

^a^ Data are presented as number (percentage) of patients unless otherwise indicated.

### Primary Outcome

The occurrence of the composite outcome (desaturation and hypotension events) was observed in 27 of 331 patients (8.2%) in the esketamine group and 69 of 329 patients (21.0%) in the placebo group (risk difference, −12.8 percentage points; OR, 0.34; 95% CI, 0.21-0.54; *P* < .001) (Table 2). Even after adjustment for baseline covariates and trial sites, the esketamine group still showed a significantly lower odds of the composite outcome (adjusted for age, BMI, smoking status, hypertension, diabetes, and trial sites: OR, 0.35; 95% CI, 0.21-0.57; *P* < .001; adjusted for sex, hypertension, procedure time, and trial sites: OR, 0.34; 95% CI, 0.21-0.55; *P* < .001; adjusted for sex and trial sites: OR, 0.34; 95% CI, 0.21-0.55; *P* < .001).

**Table 2.** Trial Outcomes

Outcome	Patients, No. (%)		Overall			Adjusted for age, BMI, smoking status, hypertension, diabetes, and trial sites			Adjusted for sex, hypertension, procedure time, and trial sites			Adjusted for sex and trial sites		
	Esketamine (n = 331)	Placebo (n = 329)	OR (95% CI)	*P* value	*q* Value	OR (95% CI)	*P* value	*q* Value	OR (95% CI)	*P* value	*q* Value	OR (95% CI)	*P* value	*q* Value
**Primary outcome**														
Composite of desaturation and hypotension	27 (8.2)	69 (21.0)	0.34 (0.21 to 0.54)	<.001	NA	0.35 (0.21 to 0.57)	<.001	NA	0.34 (0.21 to 0.55)	<.001	NA	0.34 (0.21 to 0.55)	<.001	NA
**Secondary outcomes^a^**														
Desaturation	12 (3.6)	31 (9.4)	0.36 (0.18 to 0.72)	.004	.01	0.37 (0.18 to 0.78)	.009	.03	0.41 (0.20 to 0.83)	.01	.04	0.40 (0.20 to 0.80)	.009	.03
Hypotension	16 (4.8)	44 (13.4)	0.33 (0.18 to 0.60)	<.001	<.001	0.34 (0.19 to 0.62)	<.001	<.001	0.32 (0.17 to 0.58)	<.001	<.001	0.32 (0.17 to 0.58)	<.001	<.001
Total propofol dose, median (IQR), mg^b^	130 (110 to 160)	200 (160 to 225)	−58.9 (−65.7 to −52.2)	<.001	<.001	−57.7 (−64.2 to −51.2)	<.001	<.001	−59.6 (−65.4 to −53.7)	<.001	<.001	−56.6 (−63.2 to −50.1)	<.001	<.001
NRS pain score ≥1														
Emergence from sedation	13 (3.9)	6 (1.8)	2.20 (0.83 to 5.86)	.12	.21	2.31 (0.85 to 6.28)	.10	.18	2.17 (0.80 to 5.87)	.13	.23	2.15 (0.80 to 5.78)	.13	.22
15 min later	13 (3.9)	6 (1.8)	2.20 (0.83 to 5.86)	.12	.21	2.31 (0.85 to 6.28)	.10	.18	2.17 (0.80 to 5.87)	.13	.23	2.15 (0.80 to 5.78)	.13	.22
NRS fatigue score ≥4														
Emergence from sedation	180 (54.4)	195 (59.3)	0.82 (0.60 to 1.12)	.21	.31	0.79 (0.57 to 1.09)	.14	.23	0.79 (0.57 to 1.09)	.15	.25	0.79 (0.57 to 1.08)	.14	.22
15 min later	3 (0.9)	10 (3.0)	0.29 (0.08 to 1.07)	.06	.16	0.27 (0.07 to 1.01)	.05	.14	0.27 (0.07 to 1.01)	.05	.13	0.29 (0.08 to 1.07)	.06	.16
Dizziness or headache	73 (22.1)	69 (21.0)	1.07 (0.74 to 1.55)	.74	.96	1.04 (0.71 to 1.51)	.85	>.99	1.14 (0.78 to 1.66)	.51	.66	1.09 (0.75 to 1.58)	.65	.85
Hallucination or nightmare	4 (1.2)	1 (0.3)	4.01 (0.45 to 36.10)	.22	.31	4.21 (0.46 to 38.40)	.20	.29	4.51 (0.49 to 41.10)	.18	.26	4.24 (0.47 to 38.40)	.20	.29
Nausea or vomiting	6 (1.8)	6 (1.8)	0.99 (0.32 to 3.11)	>.99	>.99	0.94 (0.30 to 2.99)	.92	>.99	0.91 (0.28 to 2.88)	.87	>.99	0.90 (0.28 to 2.86)	.86	>.99
Patients highly satisfied	325 (98.2)	329 (100)	NA	>.99	>.99	NA	>.99	>.99	NA	>.99	>.99	NA	>.99	>.99
Endoscopists highly satisfied	331 (100)	326 (99.1)	NA	>.99	>.99	NA	>.99	>.99	NA	>.99	>.99	NA	>.99	>.99

Abbreviations: BMI, body mass index; NA, not applicable; NRS, numerical rating scale; OR, odds ratio.

^a^ For secondary outcomes, multiple comparisons were corrected using the Benjamini-Hochberg approach to control for false discovery (a *q* < .05 was applied).

^b^ Values are mean difference (95% CI).

### Secondary Outcomes

For the 2 separate outcomes of desaturation and hypotension, the use of esketamine demonstrated significant reductions in the rates of desaturation (3.6% vs 9.4%; OR, 0.36; 95% CI, 0.18-0.72; *q* = .01) and hypotension (4.8% vs 13.4%; OR, 0.33; 95% CI, 0.18-0.60; *q* < .001) (Table 2). After adjustment for covariates and trial sites, the esketamine group continued to exhibit a significantly lower odds of desaturation (adjusted for age, BMI, smoking status, hypertension, diabetes, and trial sites: OR, 0.37; 95% CI, 0.18-0.78; *q* = .03; adjusted for sex, hypertension, procedure time, and trial sites: OR, 0.41; 95% CI, 0.20-0.83; *q* = .04; adjusted for sex and trial sites: OR, 0.40; 95% CI, 0.20-0.80; *q* = .03) and hypotension (adjusted for age, BMI, smoking status, hypertension, diabetes, and trial sites: OR, 0.34; 95% CI, 0.19-0.62; *q* < .001; adjusted for sex, hypertension, procedure time, and trial sites: OR, 0.32; 95% CI, 0.17-0.58; *q* < .001; adjusted for sex and trial sites: OR, 0.32; 95% CI, 0.17-0.58; *q* < .001). All patients with desaturation responded to jaw extension and supplemental oxygen therapy. No patient needed artificial airway or endotracheal intubation. All hypotension events were treated with intravenous ephedrine or phenylephrine.

Use of esketamine significantly reduced the total dose of propofol when analyzing unadjusted data (MD, −58.9 mg; 95% CI, −65.7 to −52.2 mg; *q* < .001) and after adjustment (adjusted for age, BMI, smoking status, hypertension, diabetes, and trial sites: MD, −57.7 mg; 95% CI, −64.2 to −51.2 mg; *q* < .001; adjusted for sex, hypertension, procedure time, and trial sites: MD, −59.6 mg; 95% CI, −65.4 to −53.7 mg; *q* < .001; adjusted for sex and trial sites: MD, −56.6 mg; 95% CI, −63.2 to −50.1 mg; *q* < .001). There were no significant between-group differences regarding other secondary outcomes. No patients experienced dissociation, emergence delirium, or illusions during the study.

### Subgroup Analyses

**Figure 2.**
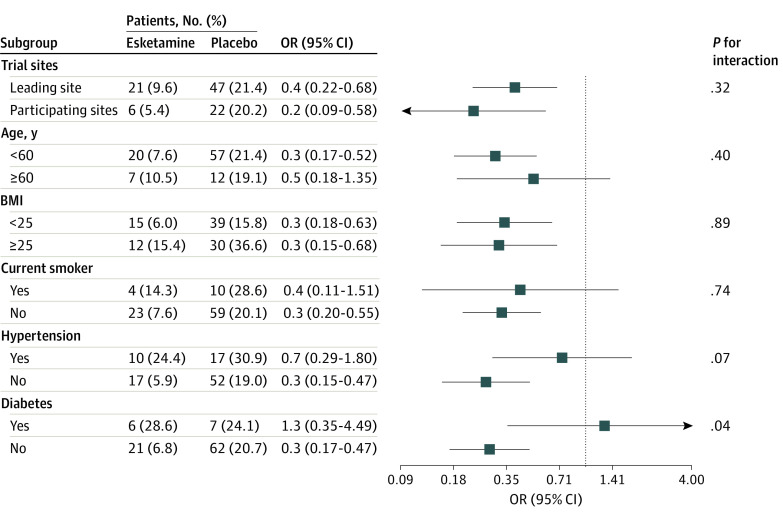
Subgroup Analyses of the Composite Outcome of Desaturation and Hypotension BMI indicates body mass index (calculated as weight in kilograms divided by height in meters squared); OR, odds ratio.

For the composite of desaturation and hypotension events, no significant heterogeneities were found among the subgroups in terms of study sites (leading vs participating), age (<60 vs ≥60 years), BMI (<25 vs ≥25), current smoker status (yes vs no), and history of hypertension (yes vs no) (Figure 2). However, significant heterogeneity was observed within the subgroup of patients with diabetes.

## Discussion

This multicenter randomized clinical trial found significant benefits of administering low-dose esketamine as an adjuvant to propofol-based sedation for patients undergoing same-visit bidirectional endoscopy. The addition of esketamine reduced the composite and separate outcomes of desaturation and hypotension during the procedures. Moreover, esketamine administration decreased propofol requirements without increasing associated adverse effects. To our knowledge, this is the first multicenter randomized clinical trial to establish the advantages of using low-dose esketamine in combination with propofol sedation for same-visit bidirectional endoscopic procedures.

During propofol sedation, hemodynamic and respiratory depression is a common concern. In previous studies involving patients undergoing gastrointestinal endoscopy with propofol sedation, the incidence rates of desaturation and hypotension events were approximately 30%.^20,22^ Interestingly, in our study, the placebo group experienced a lower rate of the composite outcome (21%), which could be attributed to low-dose sufentanil (0.1 μg/kg) to reduce propofol requirements. The combination of sufentanil and propofol for sedation is the standard practice at our study institutions and is also in line with a recent national survey in China, highlighting the preference for fentanyl or sufentanil as adjuvants to propofol sedation for gastrointestinal endoscopy.^23^

Previous studies have investigated the use of low-dose esketamine as an adjunct to propofol for sedation during endoscopic procedures. Eberl et al^22^ enrolled 162 patients undergoing endoscopic retrograde cholangiopancreatography and demonstrated that esketamine (0.15 mg/kg) led to reduced propofol requirements compared with alfentanil (2 μg/kg), without affecting recovery time or adverse events. Another study,^24^ which involved 114 patients with obesity undergoing gastroscopy, found that the combination of propofol and esketamine (0.25 mg/kg) resulted in a shorter induction time and awakening time, lower propofol consumption, more stable hemodynamics, and a reduced incidence of adverse events compared with propofol alone. Specifically, the incidence of hypoxemia was 17.3% in the esketamine group vs 40.4% in the control group, and the incidence of hypotension was 7.7% in the esketamine group vs 23.1% in the control group. These incidences of patients with obesity (mean BMI, 31-32) were higher than in the current study. Esketamine, through its activation of the sympathetic nervous system, maintains blood pressure and counters respiratory depression.^14,25,26^ Consequently, esketamine emerges as an ideal adjuvant to propofol, offering improved sedation and safety. However, prior studies had limited sample sizes, and the primary focus was not on essential patient outcomes.

In contrast, our study results are based on a robust multicenter, randomized design, providing substantial clinical evidence supporting the administration of esketamine in combination with propofol sedation for endoscopic procedures. In our patient cohort, adding low-dose esketamine (0.15 mg/kg) to propofol-based sedation significantly reduced desaturation and hypotension events by 61% (an absolute reduction of 12.8 percentage points). Furthermore, the total propofol consumption was notably reduced by 30% (an absolute reduction of 58.9 mg). These beneficial treatment effects of esketamine remained significant even after adjusting for possible confounding covariates. Notably, low-dose esketamine demonstrated a favorable safety profile because it did not increase adverse effects, such as dizziness, headache, nausea, vomiting, nightmares, hallucinations, dissociation, emergence delirium, or illusions.

### Limitations

This study has several limitations that should be acknowledged. First, we administered low-dose esketamine in combination with propofol and sufentanil to achieve adequate efficacy and safety in our patients. However, the optimal sedation regimen for endoscopic procedures is an area that still requires further investigation. Therefore, future research should continue exploring and refining the most effective and safe sedation protocols. Second, the subgroup analysis revealed a significant heterogeneity between patients with and without diabetes. However, this finding may have been influenced by the relatively small number of patients with diabetes included in the analysis. To validate and draw more robust conclusions from this result, additional studies with a larger and more balanced representation of patients with diabetes are warranted. Third, this trial was not powered to detect differences in other adverse events. Fourth, for this study, we specifically included patients aged 18 to 70 years with an ASA physical status I or II and a BMI between 18 and 30. Although these selection criteria allowed us to observe the treatment effects of esketamine in this specific patient population, they may not fully capture the effects in other age groups, those with higher ASA classifications, or individuals with obesity. Therefore, future research should explore the use of esketamine in different patient populations to gain a comprehensive understanding of its efficacy and safety across various demographics. Despite these limitations, our study provides valuable insights into the benefits of low-dose esketamine as an adjuvant to propofol-based sedation for same-visit bidirectional endoscopy. Further research and larger trials will be essential to optimize sedation strategies, confirm the subgroup findings, and extend the applicability of esketamine to broader patient populations undergoing endoscopic procedures.

## Conclusions

This multicenter randomized clinical trial provides compelling evidence that the addition of low-dose esketamine to propofol-based sedation significantly reduces the incidence of composite desaturation and hypotension events during same-visit bidirectional endoscopy. Moreover, the use of esketamine led to a noteworthy decrease in propofol requirements for these procedures while causing no safety concerns. On the basis of these findings, the incorporation of esketamine as an adjuvant to propofol sedation is strongly supported for patients undergoing same-visit bidirectional endoscopy.

## Notes

Supplement 1.
Study Protocol and Statistical Plan

Supplement 2.
Data Sharing Statement
