# Supplementary material for: Effect of Esketamine Added to Propofol Sedation on Desaturation and Hypotension in Bidirectional Endoscopy: A Randomized Clinical Trial
Source: JAMA Netw Open. 2023 Dec 20;6(12):e2347886. doi: 10.1001/jamanetworkopen.2023.47886 (PMC10733809; doi:10.1001/jamanetworkopen.2023.47886)
Supplement: Supplement 1. — Study Protocol and Statistical Plan [file jamanetwopen-e2347886-s001.pdf]

Low-dose esketamine as an adjuvant to propofol sedation for same-visit bidirectional endoscopy

A multicenter randomized controlled trial

**Title**	Low-dose esketamine as an adjuvant to propofol sedation for same-visit bidirectional endoscopy
**Institution**	First Affiliated Hospital of Soochow University
**Principal Investigator**	Fu-hai Ji, MD, PhD
**Address of Principle Investigator**	Department of Anesthesiology<br>First Affiliated Hospital of Soochow University<br>188 Shizi St, Suzhou, Jiangsu, 215006, China<br>Tel: +86-13656207331<br>Email: jifuhai@hotmail.com
**Concept and Design**	Ke Peng, Fu-hai Ji
**Study Contact**	Ke Peng<br>Address: 188 Shizi Street, Suzhou, Jiangsu, 215006, China<br>Tel: +86-15962155989<br>Email: pengke@suda.edu.cn; pengke0422@163.com
**Version**	1.0
**Date**	2021-08-17

Table of Contents

1. Synopsis 4
2. Flow Chart 6
3. Glossary 7
4. Background and Rationale 9
   - 4.1 Sedation for gastrointestinal endoscopic procedures 9
   - 4.2 Use of esketamine 9
   - 4.3 The aim of this study 9
5. Study Design 10
   - 5.1 Description of this study 10
   - 5.2 Duration of this study 10
6. Study Outcomes 11
   - 6.1 Primary outcome 11
   - 6.2 Secondary outcomes 11
7. Selection of Patients 12
   - 7.1 Inclusion criteria 12
   - 7.2 Exclusion criteria 12
8. Study Treatment 13
   - 8.1 Randomization and blinding 13
   - 8.2 Sedation and study Interventions 13
   - 8.3 Accountability and compliance 14
9. Safety Concerns 15
   - 9.1 Safety profile of propofol and esketamine 15
   - 9.2 Peri-procedural monitoring 15
   - 9.3 Discontinuation of intervention and unmasking 15
10. Statistical Plan 17
    - 10.1 Sample size 17
    - 10.2 Statistical analysis 17
    - 10.3 Statisticians 18
11. Ethical Considerations 19
    - 11.1 Ethical approval and clinical trial registration 19

11.2 Informed consent **19**

11.3 Responsibilities **19**

12. Data Management and Clinical Trial Report **20**

12.1 Source documents **20**

12.2 Case Report Forms (CRFs) **20**

12.3 Data protection **20**

12.4 Clinical trial report **20**

13. References **21**

1. Synopsis

**Study Title**	Low-dose esketamine as an adjuvant to propofol sedation for same-visit bidirectional endoscopy
**Study Design**	Researcher-initiated, multicenter, randomized, placebo-controlled trial
**Principal Investigator**	Fu-hai Ji, MD, PhD
**Trial Site**	Three medical centers in eastern China<br>➢ The First Affiliated Hospital of Soochow University<br>➢ Taicang First People's Hospital<br>➢ The People's Hospital of SND
**Selection Criteria**	**Inclusion Criteria:**<br>1. 18–70 years old;<br>2. American society of Anesthesiologists status I or II;<br>3. Body mass index (BMI) 18–30 kg/m^2;<br>4. Scheduled for same-visit bidirectional endoscopy.<br>**Exclusion Criteria:**<br>1. Severe cardiovascular or pulmonary diseases;<br>2. Renal or liver dysfunction;<br>3. Neurocognitive or psychiatric disorders;<br>4. Seizures or epilepsy;<br>5. Alcoholism, preoperative use of sedatives or analgesics;<br>6. Hypersensitivity to the medications in this study.
**Study Outcomes**	**Primary outcome:**<br>Composite of desaturation (defined as SpO_2 <90%) and hypotension (defined as systolic blood pressure <80 mmHg or a decrease in systolic blood pressure >30% of baseline)
	**Secondary outcomes:**<br>— Desaturation;<br>— Hypotension;<br>— Total dose of propofol;<br>— Pain at emergence and 15 min later;<br>— Fatigue at emergence and 15 min later;<br>— Dizziness or headache;<br>— Hallucination or nightmare;<br>— Nausea or vomiting;<br>— Patient satisfaction and;<br>— Endoscopist satisfaction.

**Expected Number of Patients**	660 adult patients
**Study Drug and Interventions**	Patients will be randomized (1:1) to receive esketamine 0.15 mg/kg or normal saline placebo after induction of sedation.
**Safety Concerns**	No additional risk associated with low-dose esketamine use is expected.<br>— Esketamine may induce psychiatric adverse effects (dizziness, headache, hallucination, nightmare, dissociation, emergence delirium, or illusions), but most studies found these effects to be self-limiting and clinically benign.<br>— To avoid potential adverse effects, a low-dose esketamine will be given.
**Statistical Plan**	The treatment effects of esketamine vs placebo will be assessed using the odds ratio (OR) or difference in means/medians with 95% confidence intervals (CI). All data will be analyzed according to the modified intention-to-treat principle, including any randomized patient with their result of the primary outcome available.<br>Prespecified subgroup analyses will be conducted based on trial center, age, BMI, current smoker, history of hypertension, and history of diabetes. Neither an interim analysis nor an imputation of missing data is planned.<br>Analyses will be performed using GraphPad Prism software (version 9.00; GraphPad, San Diego, CA) and R software (version 3.6.0, R Foundation for Statistical Computing, Vienna, Austria) according to a prespecified analysis plan.
**Study Period**	From anesthesia clinic visit until hospital discharge

2. Flow Chart

	Screening	Study Phase			
		Treatment	Post treatment follow-up		
	Anesthesia clinic visit	In OR	During sedation	Emergence from sedation	Hospital discharge
Evaluation					
Inclusion/Exclusion Criteria	×				
Medical/Surgical History	×				
Informed Consent	×				
Prior Medication History	×				
Patient Demographics	×				
Clinical Examination	×				
Vital Signs	×				
Lab Testing	×				
Randomization		×			
Treatment					
Intervention		×			
Compliance		×			
Outcome evaluation					
Primary			×		
Secondary			×	×	×
Safety					
AE/SAE (if any)			×	×	×

AE, adverse event; SAE, serious adverse event.

3. Glossary

- **Good Clinical Practice (GCP)**

An international standard of ethics and scientific quality to design, conduct, register and confront studies on human subjects. Adherence to these standards not only guarantees the safety, the well-being, and the rights of the participates, in accordance with the Helsinki Declaration (1947) principles, but also the reliability of the study data.

- **Ethic Committee (EC)**

An independent organization, composed by health care and non-heath care staff that is responsible for the safeguard of the safety, the well-being, and the rights of study subjects and that must also publicly guarantee this safeguard by, for example, expressing an opinion on the experimental protocol, on the investigators, on the adequacy of the structures, methods and documents used to inform the subjects and obtain informed consent.

- **Clinical Research Office (CRO)**

The Clinical Research Office takes care of the preparation and revision of all documents involved in research protocols (whether sponsored, internal, or part of a study group), including amendments and modifications, that are then analyzed by the EC at First Affiliated Hospital of Soochow University to be approved and then authorized. The CRO works as a scientific and technical secretariat of the EC.

- **No Profit Study**

No profit study does not aim at the industrial development of a drug or, in any case, not for profit, but aims at improving clinical practice. This objective must be guaranteed by the protocol relevance, by the peculiarity of the disease, and by the type of treatment.

- **Investigator**

A physician qualified in clinical research is responsible for this clinical trial in a university hospital. If the trial is followed by a group of investigators in the same hospital, the investigator who is responsible is called “Principal Investigator” (PI).

- **Case Report Form (CRF)**

The Case Report Form (CRF) is a printed or digital document designed to register all the information required by the study protocol that must be reported regarding each study subject.

- **Adverse Event (AE)**

Any negative clinical event that involves a patient undergoing a clinical trial who has received a study drug, even though the event does not necessarily have a causative correlation with the treatment.

- **Serious Adverse Event (SAE)**

Any adverse event or drug reaction that, regardless of the dose, causes the patient's death or threatens the subject's life, requires hospitalization, or prolongs hospitalization, or causes prolonged or severe invalidity, or involved a congenital anomaly or a birth problem.

4. Background and Rationale

4.1 Sedation for gastrointestinal endoscopic procedures

Over the past decade, the volume of gastrointestinal endoscopic procedures has increased more than tenfold.[1] A recent nationwide survey showed that the overall number of gastrointestinal endoscopic procedures in China is 14 million per year.[2] This number is predicted to reach 51 million by 2030. Many patients receive esophagogastroduodenoscopy and colonoscopy in a same hospital visit (also called same-visit bidirectional endoscopy).[3,4] Its benefits include reduction in medical costs and facilitation of healthcare decision-making.[5] Sedation is also increasingly provided to improve patient comfort and facilitate the procedures.[6] Propofol alone or combined with analgesics is commonly used for sedation in endoscopic procedures, but is associated with adverse events such as desaturation and hypotension.[7-9]

4.2 Use of esketamine

Esketamine is an N-methyl-D-aspartate receptor antagonist and the dextrorotatory isomer of ketamine. Esketamine has two times more potent hypnotic and analgesic effects and less psychiatric side effects than ketamine.[10,11] Recent studies suggested that the use of a subanesthetic dose of esketamine maintained hemodynamic stability and reduced respiratory depression for surgical patients.[12] Thus, a low-dose esketamine combined with propofol may provide adequate sedation and analgesia for patients undergoing gastrointestinal endoscopic procedures.

4.3 The aim of this study

We design this multicenter randomized controlled trial to investigate the effects of a low-dose esketamine added to propofol-based sedation on desaturation and hypotension events in patients undergoing same-visit bidirectional endoscopy. We hypothesize that esketamine used as an adjuvant to propofol sedation would reduce the incidence of the composite of desaturation and hypotension during these endoscopic procedures.

5. Study Design

5.1 Description of this study

This is a researcher-initiated, multicenter, randomized, placebo-controlled study. After screening of eligibility, patients who meet the enrollment criteria will be randomized to receive 1 of 2 treatments: esketamine or normal saline placebo. The randomization results will be stored in sealed opaque envelopes.

The randomization will be performed at the last available moment, which will reduce potential biases. Both esketamine and normal saline are provided as clear aqueous solutions in identical bottles and labeled with previous set code to achieve blinding. All patients and the study personnel, including those involved in the endoscopic unit, data collection, data entry or data analysis will be blinded to treatment assignment for the duration of the study.

Consented patients will receive standard perioperative management and corresponding study interventions. After sedation induction, patients will receive either esketamine or normal saline. The hemodynamics and respiration of the patients will be monitored. Data will be collected by trained observers who will not participate in patient care and will be blinded to the administered drug. Study outcomes are assessed during the procedures and until hospital discharge.

5.2 Duration of this study

Study team physicians are responsible for screening all patients undergoing a scheduled procedure. The number of eligible, consented, enrolled, and randomized patients is recorded in addition to the reasons for non-participation in the trial. During their hospital stay, patients are closely monitored and all outcomes are recorded. For this reason, missing endpoints are expected to be rare. The day before their appointment, the patient is contacted by a member of the study staff to remind them that a member of the team will collect research data during their visit. This is done by a phone call (or email if unreachable by phone). Based on the experience and our pilot data in our institution as well as the results of previous studies, it is estimated that it will take 10 months to enroll enough patients.

6. Study Outcomes

6.1 Primary outcome

- Composite of desaturation and hypotension.

Desaturation is defined as peripheral oxygen saturation (SpO_2) <90%.

Hypotension is defined as systolic blood pressure <80 mmHg or a decrease in systolic blood pressure >30% of baseline.

6.2 Secondary outcomes

- Desaturation;
- Hypotension;
- Total dose of propofol;
- Pain at emergence and 15 min later;
- Fatigue at emergence and 15 min later;
- Dizziness or headache;
- Hallucination or nightmare;
- Nausea or vomiting;
- Patient satisfaction and;
- Endoscopist satisfaction.

7. Selection of Patients

7.1 Inclusion criteria

- 18–70 years old;
- American society of Anesthesiologists status I or II;
- Body mass index (BMI) 18–30 kg/m^2;
- Scheduled for same-visit bidirectional endoscopy.

7.2 Exclusion criteria

- Severe cardiovascular or pulmonary diseases;
- Renal or liver dysfunction;
- Neurocognitive or psychiatric disorders;
- Seizures or epilepsy;
- Alcoholism, preoperative use of sedatives or analgesics;
- Hypersensitivity to the medications in this study.

8. Study Treatment

8.1 Randomization and blinding

A biostatistician who is independent of data management and analyses generates the randomization list using an online tool (https://www.sealedenvelope.com/simple-randomiser/v1/lists). The randomization is performed with an allocation ratio of 1:1, permuted blocks of 2 and 4, and stratification by study center. The details of group allocation will be sealed in opaque envelopes until the end of the study. Based on the randomization list, an independent research nurse in each center will distribute the study medications (esketamine or normal saline placebo) in identical syringes labeled with study numbers only. It is impossible to distinguish the content in the syringes, because both esketamine and normal saline are clear and colorless. To avoid potential interference with blinding, esketamine or normal saline will be administered immediately after the induction of sedation. Patients, anesthesiologists, endoscopists, care providers, and outcome observers will all remain masked to the group assignment until the end of final analysis.

In case of an emergency (e.g., unexpected rapid deterioration in the patient's clinical status), attending anesthesiologists or endoscopists could request unmasking of the treatment allocation, or adjust drug administration if necessary. To maintain the overall quality, legitimacy, and integrity of the clinical trial, unblinding of the test drug may occur only in critical circumstances when severe adverse events happen and considered to be related to esketamine administration. In this circumstance, the PI. fully documents and explains the reasons for unblinding in a report to the Institutional Review Board (IRB).

8.2 Sedation and study interventions

All patients will undergo bidirectional endoscopy in the esophagogastroduodenoscopy-colonoscopy sequence. Throughout the procedures, heart rate and peripheral oxygen saturation (SpO_2) will be continuously monitored, and noninvasive blood pressure will be measured at 3-min intervals. All patients will receive supplemental oxygen at a flow of 3 L/min through a nasal cannula. A standardized intravenous sedation will be provided by the same anesthesiologist in each study center. Intravenous sufentanil 0.1 μg/kg and propofol 0.5 mg/kg will be administered for induction of sedation. Thereafter, the esketamine group will receive intravenous esketamine 0.15 mg/kg, while the normal saline group will receive

the same volume of normal saline. Propofol will be titrated with additional doses of 0.2–0.3 mg/kg to reach the target sedation levels or to treat discomfort responses (such as grimaces, gag, moan, or body movement). The level of sedation will be assessed using the Modified Observer's Alertness/Sedation scale (MOAA/S) every 30 seconds (see below).[13,14] At the beginning of esophagogastroduodenoscopy, the target sedation level will be MOAA/S score = 1. Subsequently and during the colonoscopy, the target sedation level will be MOAA/S score = 2. Following the procedures, patients will be transferred to a recovery room. Patients are ready for discharge when they are fully awake with a modified Aldrete score of 10.[4]

Modified Observer's Alertness/Sedation scale (MOAA/S)

MOAA/S score	Responsiveness	ASA continuum of sedation
5	Prompt response to name spoken in a normal tone	Minimal sedation
4	Lethargic response to name spoken in a normal tone	Moderate sedation
3	Response only to name called loudly or repeatedly	Moderate sedation
2	Response only to mild prodding or shaking	Moderate sedation
1	Response only to painful stimulus (trapezius squeeze)	Deep sedation
0	No response to painful stimulus (trapezius squeeze)	General anesthesia

8.3 Accountability and compliance

This study is primarily designed to see the effects of low-dose esketamine added to propofol-based sedation on desaturation and hypotension in adult patients undergoing same-visit bidirectional endoscopy. The principal investigator will be responsible that this trial is conducted as specified and in accordance with the applicable regulatory requirements. Jiangsu Hengrui Medicine Co, Ltd, Jiangsu, China helps to provide the blinded intervention drugs and placebos, but the pharmaceutical company will not participate in any other parts of the trial. All patients will receive standard perioperative treatment and the investigational drug will be delivered according to this prespecified standard protocol. The name and dosage of other relevant medications will be recorded in the Case Report Form.

9. Safety Concerns

9.1 Safety profile of propofol and esketamine

Propofol is the most popular sedation drug for gastrointestinal endoscopic procedures. Compared with the traditional sedation with benzodiazepines such as midazolam or diazepam, sedation with propofol for digestive endoscopy leads to a shorter time to sedation, a faster recovery profile, and improved patient satisfaction.[9] Despite these benefits, propofol use for sedation can cause cardiorespiratory complications. Studies showed that the incidence of these complications ranged from 20% to 60%, depending on the type of procedures and choice of sedation regimens.[9,15-18]

Ketamine or esketamine can be used for sedation, alone or as an adjuvant to propofol. Esketamine, a novel N-methyl-D-aspartate receptor antagonist, has a 2-fold higher sedative potency and a lower risk of side effects than racemic ketamine.[10,11] The role of esketamine in endoscopic procedures is not fully understood. For our patients, intravenous propofol 0.5 mg/kg in combination with sufentanil 0.1 μg/ml will be used for sedation induction, followed by propofol titration to the target sedation levels. This strategy will minimize the risk of oversedation and associated complications.

9.2 Peri-procedural monitoring

Throughout the procedures, heart rate and peripheral oxygen saturation (SpO_2) will be continuously monitored, and noninvasive blood pressure will be measured at 3-min intervals. All patients will receive supplemental oxygen at a flow of 3 L/min through a nasal cannula. A standardized intravenous sedation will be provided by the same anesthesiologist at each study center. All patients will undergo bidirectional endoscopy in the esophagogastroduodenoscopy-colonoscopy sequence. Following the procedures, patients will be transferred to a recovery room. Patients are ready for discharge when they are fully awake and a modified Aldrete score of 10 is reached.[4]

9.3 Discontinuation of intervention and unmasking

The criteria for patient discontinuation from the study are as follows:

1) Voluntary discontinuation by a patient;

2) Exiting the protocol for safety reasons based on the judgement of the clinical or research staff including acute worsening of vital signs.

In case of an emergency (e.g., unexpected rapid deterioration in the patient's clinical status), attending anesthesiologists or endoscopist could request unmasking of the treatment allocation if necessary. To maintain the overall quality, legitimacy, and integrity of the clinical trial, unblinding of the test drug may occur only in critical circumstances when severe adverse events happen and considered to be related to esketamine administration. In this circumstance, the PI. fully documents and explains the reasons for unblinding in a report to the IRB.

10. Statistical Plan

10.1 Sample size

Our pilot results showed that 7 patients (21.9%) developed desaturation and 5 patients (15.6%) experienced hypotension during same-visit bidirectional endoscopy. These incidences are in line with the recent literature.[15,16] We use a conservative composite incidence of 30% for the power analysis of this multicenter trial. We hypothesize that the addition of a low-dose esketamine would reduce the composite incidence of desaturation and hypotension to 20% (i.e., an absolute reduction rate of 10%). Based on this assumption, 294 patients will be required in each group with a power of 80% at an α level of 0.05. Considering a potential dropout rate of 10%, we decide to recruit a total of 660 patients, with 330 in each group. The sample size is calculated using the PASS software (version 11.0.7, NCSS, LCC, Kaysville, UT, USA).

10.2 Statistical analysis

All data analyses will be carried out according to the pre-established analysis plan. Continuous variables will be tested for normal distribution using the Shapiro-Wilk test and presented as means ± standard deviations if normally distributed and medians (interquartile ranges) if not. The groups will be compared using the independent t-test and Mann–Whitney rank-sum test, as appropriate. Categorical variables will be presented as numbers (percentages) and analyzed using the Chi-squared test or Fisher exact test, as appropriate.

Demographic data and baseline characteristics will be presented using descriptive statistics only. For the primary and secondary endpoints, the therapeutic effect between the two study groups will be assessed using the odds ratio (OR) or difference in means/medians with 95% confidence intervals (CI). Considering multiple comparison corrections for the secondary endpoints, the Benjamini-Hochberg approach will be applied to control for false discovery. In addition, these endpoints will be analyzed using the multivariate logistic regression model or the generalized linear model adjusting for baseline covariates (age, BMI, smoking status, history of hypertension, and history of diabetes) and study center. The P values before and after adjustment will be presented.

In addition, subgroup analyses based on the primary endpoint will be carried out to further investigate whether the study interventions would produce different effects in the

follow subgroups: age (< 60 years vs. ≥ 60 years), BMI (< 25 kg/m^2 vs. ≥ 25 kg/m^2), current smoker (yes vs. no), history of hypertension (yes vs. no), and history of diabetes (yes vs. no). The interaction analysis across the subgroups will be performed using a logistic regression model.

All analyses will be performed on the intention-to-treat basis. We expect that protocol violation would be uncommon in this study. We have no plans for an interim analysis or imputation of missing data. All statistical tests will be two-sided and $P < 0.05$ indicates a statistically significant difference. Statistical analyses will be performed using the GraphPad Prism software (version 9.00; GraphPad, San Diego, CA) and R software (version 3.6.0, R Foundation for Statistical Computing, Vienna, Austria).

10.3 Statisticians

Trial statistical analysis: Yu-qin Long, Ke Peng

Independent statistician: Yao-yu Ying (Department of Epidemiology and Biostatistics, School of Public Health, Medical College of Soochow University, Suzhou, Jiangsu, 215123, China)

11. Ethical Considerations

11.1 Ethical approval and clinical trial registration

The Ethics Committee of The First Affiliated Hospital of Soochow University will assess the protocol and the written and dated approval signed by the Ethics Committee chairman will be obtained. The study will be registered at the Chinese Clinical Trial Registry (www.chictr.org.cn). This study will be conducted in accordance with the principles laid down by the World Medical Assembly and all applicable amendments (Helsinki, 1964) and the ICH guidelines for Good Clinical Practice. This clinical trial will be conducted in compliance with international laws and regulations, and laws and regulations of China, as well as any applicable guidelines.

11.2 Informed consent

The investigator (according to applicable regulatory requirements), or a person designated by the investigator, and under the investigator's responsibility, will fully inform the patient of all pertinent aspects of the clinical trial. All participants will be informed to the most fully extent about this study, in languages and terms they are able to understand. Prior to a patient's participation in the clinical trial, he or she MUST sign the written Informed Consent Form. It will also be made clear to the patient that he or she can withdraw from the study at any time without giving reasons and that they will not be in any way disadvantaged by this. Any Informed Consent will be retained by the Investigator. A copy of the signed and dated written Informed Consent Form will be provided to the patient.

11.3 Responsibilities

The investigators should perform the clinical trial in accordance with this clinical trial protocol, ICH/Good Clinical Practice, and the applicable regulatory requirements. The investigators ensure compliance with all procedures required by the clinical trial protocol and with all study required procedures. The investigator agrees to provide all information requested in the Case Report Forms (CRFs) in an accurate and legible manner.

12. Data Management and Clinical Trial Report

12.1 Source documents

According to the ICH/Good Clinical Practice, the monitoring team must check the Case Report Form (CRFs) entries and the source documents.

12.2 Case Report Forms (CRFs)

It is the responsibility of the investigators to maintain adequate and accurate CRFs records. All CRFs will be completed electronically in their entirety to ensure accurate interpretation of data.

12.3 Data protection

Data will be stored in the electronic database without indicating the name of the patients (a numeric code will be used).

12.4 Clinical trial report

The principal investigator will be responsible for preparing a clinical trial report. When all data have been fully analyzed, the results of the clinical trial will be communicated to all investigators and to the Competent Authority.

13. References

1. Lucendo AJ, González-Huix F, Tenias JM, et al. Gastrointestinal endoscopy sedation and monitoring practices in Spain: a nationwide survey in the year 2014. *Endoscopy.* 2015;47(4):383-390.
2. Zhou S, Zhu Z, Dai W, et al. National survey on sedation for gastrointestinal endoscopy in 2758 Chinese hospitals. *British journal of anaesthesia.* 2021;127(1):56-64.
3. Cao Y, Yang J, Li J, et al. Comparison of procedural sequences in same-day painless bidirectional endoscopy: Single-center, prospective, randomized study. *Dig Endosc.* 2017;29(3):330-337.
4. Laoveeravat P, Thavaraputta S, Suchartlikitwong S, et al. Optimal sequences of same-visit bidirectional endoscopy: Systematic review and meta-analysis. *Dig Endosc.* 2020;32(5):706-714.
5. Urquhart J, Eisen G, Faigel DO, Mattek N, Holub J, Lieberman DA. A closer look at same-day bidirectional endoscopy. *Gastrointest Endosc.* 2009;69(2):271-277.
6. Dossa F, Megetto O, Yakubu M, Zhang DDQ, Baxter NN. Sedation practices for routine gastrointestinal endoscopy: a systematic review of recommendations. *BMC gastroenterology.* 2021;21(1):22.
7. Ogawa T, Tomoda T, Kato H, Akimoto Y, Tanaka S, Okada H. Propofol sedation with a target-controlled infusion pump in elderly patients undergoing ERCP. *Gastrointestinal endoscopy.* 2020;92(2):301-307.
8. Friedrich-Rust M, Welte M, Welte C, et al. Capnographic monitoring of propofol-based sedation during colonoscopy. *Endoscopy.* 2014;46(3):236-244.
9. Zhang W, Zhu Z, Zheng Y. Effect and safety of propofol for sedation during colonoscopy: A meta-analysis. *J Clin Anesth.* 2018;51:10-18.
10. Molero P, Ramos-Quiroga JA, Martin-Santos R, Calvo-Sánchez E, Gutiérrez-Rojas L, Meana JJ. Antidepressant Efficacy and Tolerability of Ketamine and Esketamine: A Critical Review. *CNS drugs.* 2018;32(5):411-420.
11. Kamp J, Jonkman K, van Velzen M, et al. Pharmacokinetics of ketamine and its major metabolites norketamine, hydroxynorketamine, and dehydronorketamine: a model-based analysis. *British journal of anaesthesia.* 2020;125(5):750-761.
12. Jonkman K, van Rijnsoever E, Olofsen E, et al. Esketamine counters opioid-induced respiratory depression. *British journal of anaesthesia.* 2018;120(5):1117-1127.
13. Barends CRM, Driesens MK, Struys M, Visser A, Absalom AR. Intranasal dexmedetomidine in elderly subjects with or without beta blockade: a randomised double-blind single-ascending-dose cohort study. *British journal of anaesthesia.* 2020.

14. Kashiwagi K, Hosoe N, Takahashi K, et al. Prospective, randomized, placebo-controlled trial evaluating the efficacy and safety of propofol sedation by anesthesiologists and gastroenterologist-led teams using computer-assisted personalized sedation during upper and lower gastrointestinal endoscopy. *Dig Endosc.* 2016;28(6):657-664.
15. Eberl S, Koers L, van Hooft J, et al. The effectiveness of a low-dose esketamine versus an alfentanil adjunct to propofol sedation during endoscopic retrograde cholangiopancreatography: A randomised controlled multicentre trial. *European journal of anaesthesiology.* 2020;37(5):394-401.
16. Chiang MH, Wu SC, You CH, et al. Target-controlled infusion vs. manually controlled infusion of propofol with alfentanil for bidirectional endoscopy: a randomized controlled trial. *Endoscopy.* 2013;45(11):907-914.
17. Forster C, Vanhaudenhuyse A, Gast P, et al. Intravenous infusion of lidocaine significantly reduces propofol dose for colonoscopy: a randomised placebo-controlled study. *British journal of anaesthesia.* 2018;121(5):1059-1064.
18. Yoon SW, Choi GJ, Lee OH, et al. Comparison of propofol monotherapy and propofol combination therapy for sedation during gastrointestinal endoscopy: A systematic review and meta-analysis. *Dig Endosc.* 2018;30(5):580-591.
