# Supplementary material for: Effect of Esketamine Added to Propofol Sedation on Desaturation and Hypotension in Bidirectional Endoscopy: A Randomized Clinical Trial
Source: JAMA Netw Open. 2023 Dec 20;6(12):e2347886. doi: 10.1001/jamanetworkopen.2023.47886 (PMC10733809; doi:10.1001/jamanetworkopen.2023.47886)
Supplement: Supplement 2. — Data Sharing Statement [file jamanetwopen-e2347886-s002.pdf]

Data Sharing Statement

Song. Effect of Esketamine Added to Propofol Sedation on Desaturation and Hypotension in Bidirectional Endoscopy. *JAMA Netw Open*. Published December 20, 2023.
doi:10.1001/jamanetworkopen.2023.47886

Data
Data available: Yes
Data types: Deidentified participant data
How to access data: The data that support the findings of this study are available from the corresponding author upon reasonable request. Ke Peng: pengke0422@163.com
When available: With publication

Supporting Documents
Document types: None

Additional Information
Who can access the data: researchers whose proposed use of the data has been approved
Types of analyses: for any purpose
Mechanisms of data availability: after approval of a proposal
